# Elucidating the Substrate Envelope of Enterovirus 68-3C Protease: Structural Basis of Specificity and Potential Resistance

**DOI:** 10.3390/v16091419

**Published:** 2024-09-05

**Authors:** Vincent N. Azzolino, Ala M. Shaqra, Akbar Ali, Nese Kurt Yilmaz, Celia A. Schiffer

**Affiliations:** Department of Biochemistry and Molecular Biotechnology, University of Massachusetts Chan Medical School, Worcester, MA 01605, USA; vincent.azzolino@umassmed.edu (V.N.A.); ala.shaqra@umassmed.edu (A.M.S.); akbar.ali@umassmed.edu (A.A.); nese.kurtyilmaz@umassmed.edu (N.K.Y.)

**Keywords:** enterovirus, EV68, substrate recognition, protease, drug resistance, protein structure, molecular modeling

## Abstract

Enterovirus-D68 (EV68) has emerged as a global health concern over the last decade with severe symptomatic infections resulting in long-lasting neurological deficits and death. Unfortunately, there are currently no FDA-approved antiviral drugs for EV68 or any other non-polio enterovirus. One particularly attractive class of potential drugs are small molecules inhibitors, which can target the conserved active site of EV68-3C protease. For other viral proteases, we have demonstrated that the emergence of drug resistance can be minimized by designing inhibitors that leverage the evolutionary constraints of substrate specificity. However, the structural characterization of EV68-3C protease bound to its substrates has been lacking. Here, we have determined the substrate specificity of EV68-3C protease through molecular modeling, molecular dynamics (MD) simulations, and co-crystal structures. Molecular models enabled us to successfully characterize the conserved hydrogen-bond networks between EV68-3C protease and the peptides corresponding to the viral cleavage sites. In addition, co-crystal structures we determined have revealed substrate-induced conformational changes of the protease which involved new interactions, primarily surrounding the S1 pocket. We calculated the substrate envelope, the three-dimensional consensus volume occupied by the substrates within the active site. With the elucidation of the EV68-3C protease substrate envelope, we evaluated how 3C protease inhibitors, AG7088 and SG-85, fit within the active site to predict potential resistance mutations.

## 1. Introduction

Enteroviruses, belonging to the Picornaviridae family, are small, positive-sense, single-stranded, non-enveloped RNA viruses that cause a variety of illnesses such as polio, respiratory disease, encephalitis, myocarditis, hepatitis, hand-foot-and-mouth disease (HFMD), as well as the common cold [1]. Enteroviruses mainly infect young children and immunocompromised individuals [2,3,4], and while most cause self-limiting disease, certain strains of non-polio enteroviruses, such as enterovirus 68 (EV68), can cause severely debilitating and long-lasting neurological deficits due to their neurotropism [2,5,6,7]. EV68 has been associated with severe respiratory symptoms as well as acute flaccid myelitis (AFM) [8,9], a polio-like paralysis with debilitating muscle weakness, where more than 90% of patients never reach full neurologic recovery Reviewed in Refs. [10,11,12,13,14]. The 2014 EV68 outbreak across the United States caused over 1100 cases of severe respiratory illness and the deaths of fourteen children [2,5]. Additional outbreaks were recorded in 2016 and 2018 with increased incidence and severity as well as increased case reports of AFM associated with EV68 [8,9,10,15,16]. The trend of outbreaks for this debilitating and lethal enterovirus strain is cause for global concern and requires attention to develop effective therapeutics.

**Figure 1:**
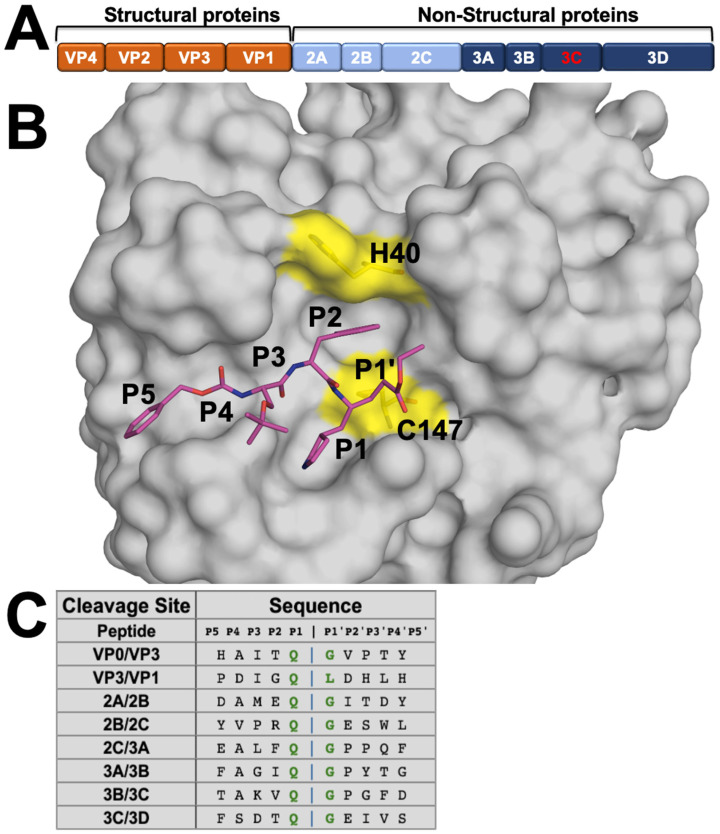
EV68-3C protease substrates and structure. (**A**) EV68 polyprotein prior to cleavage by the 3C protease into structural and non-structural viral proteins. (**B**) Crystal structure of EV68-3C protease in surface representation bound to a peptidomimetic inhibitor (SG-85, in magenta sticks) with cysteine protease’s catalytic dyad residues (H40 and C147) highlighted in yellow (PDB ID: 3ZVF). The inhibitor is labeled with corresponding P5 to P1′ moieties. (**C**) Amino acid sequences of EV68-3C protease cleavage sites in the viral polyprotein, with a conserved glutamine (Q) in the P1 position, generally followed by a glycine (G) at P1′. The sequences are highly diverse distal to the cut site (denoted by the blue vertical bar).

There are currently no U.S. Federal Drug Administration (FDA)-approved therapies that effectively treat EV68, or any other non-polio EVs [5,17]. Direct-acting antivirals (DAAs) are effective treatment options for viral infections and work by targeting essential steps in the viral lifecycle, such as the proteases responsible for polyprotein processing [1,5,18]. Inhibitors targeting viral proteases have proven to be effective antivirals that can advance to clinic [19], as most recently with nirmatrelvir for SARS-CoV-2 3C-like main protease [20]. During viral replication of EV68, the viral genome is translated into a single, large polyprotein comprised of structural and non-structural proteins (Figure 1A) [1,5,21]. The viral polyprotein needs to be cleaved into individual proteins for viral replication to proceed. The 3C protease cleaves eight sites in the polyprotein with a preference for a glutamine–glycine (QG) motif at the P1/P1′ positions (Figure 1C) [1,21]. The conserved essential function in viral replication makes the 3C protease a promising target for DAA development.

Viral proteases often cleave many substrates within the viral polyprotein of diverse sequences, suggesting recognition is not entirely determined by sequence. We determined that the shape the substrates with diverse sequences adopt, which we called the “substrate envelope”, defines the basis of substrate specificity [22]. We have established the substrate envelope approach for viral proteases of HIV-1, HCV, and most recently SARS-CoV-2 [23,24,25,26]. The substrate envelope also predicts the emergence of drug resistance mutations to specific inhibitors. Inhibitors extending beyond the substrate envelope are more susceptible to drug resistance mutations, while inhibitors that fit within the volume exhibit higher barriers to resistance [22,25,27]. When inhibitors fit within the substrate envelope, mutations are less likely to occur as changes that negatively affect inhibitor binding simultaneously would impair the protease’s ability to recognize substrates, decreasing the likelihood of viable resistance mutations [28,29,30,31]. Although EV68-3C protease substrates have the conserved QG P1/P1′ cleavage motif, their sequences are diverse distal to the scissile bond where cleavage occurs. Therefore, characterizing the EV68-3C protease substrate envelope would enable assessing inhibitor susceptibility to potential resistance and guide the design of robust antivirals with lower susceptibility to drug-resistant variants.

The efficacy of various inhibitors targeting enteroviral 3C proteases [17,19,21,32,33,34,35,36,37,38,39,40,41], as well as inhibitors targeting the 3C-like protease of SARS-CoV-2, have been previously reported [20]. Some of these inhibitors have been further characterized by protein crystallography, bound within the active site of EV68-3C protease [17,42]. However, how inhibitor binding compares to that of natural viral substrates and the substrate envelope of enteroviral 3C proteases has remained elusive. Elucidation of the substrate envelope will reveal the intermolecular interactions essential for molecular recognition and evaluate the susceptibility of current inhibitors to potential resistance mutations to better inform rational and structural-guided drug design [23,29,31,43,44,45,46]. A collection of three mutations within 3C proteases of human rhinovirus (HRV) have been reported to cause up to a 15-fold decrease in the potency of inhibitory compounds, as was demonstrated in HRV2-3C protease targeted with rupintrivir [47]. Here, we report the substrate envelope of EV68-3C protease determined by structural analysis of peptides corresponding to all eight viral polypeptide cleavage sites bound to the protease. The substrate envelope was calculated through rigorous molecular modeling with subsequent molecular dynamics (MD) simulations and validation to define the consensus volume occupied by the substrates at the active site, as has been previously conducted for HIV-1 and HCV proteases [48,49]. This approach was further validated by the first co-crystal structure of EV68-3C with a native viral substrate peptide. We also determined a high-resolution apo structure of the inactive C147A mutant EV68-3C protease for comparison. Our analysis here provides a structural understanding of the intermolecular interactions with viral substrates and changes in protease conformation upon substrate binding. In addition, elucidation of the EV68-3C protease substrate envelope provides critical insights for designing inhibitors which are robust against mutations that can confer drug resistance.

## 2. Materials and Methods

### 2.1. Expression and Purification of EV68-3C Protease

His-tagged EV68-3C protease (WT and C147A mutant) with an embedded thrombin cleavage site was cloned into a pET28-a vector. Each vector was transformed into Rosetta BL21(DE3) *E. coli* cells using standard techniques. Overnight cultures in TB + kanamycin media were grown from single colonies. Each culture was used to inoculate 6 × 1 L cultures in TB, supplemented with 4% glycerol and 35 µg/mL kanamycin. These cultures grew in Fernbach flasks at 37 °C while shaking at 180 rpm, until the OD600 reached approximately 0.75, at which point the temperature was reduced to 18 °C, 1 mM IPTG was added, and they were left to grow overnight.

For both the WT and inactive C147A mutant, cell pellets were resuspended in EVNi1A Buffer (50 mM HEPES pH 7.5, 750 mM NaCl, 10 mM imidazole, 10% glycerol *w*/*v*) prior to lysis by three passes through a cell disruptor/homogenizer at 80 psi. Cell lysate was then clarified by centrifugation at 25,000× *g* for 1 h. A 5 mL His-Trap crude FF column (Cytiva, Marlborough, MA, USA) was pre-equilibrated with EVNi1A Buffer using an AKTA FPLC. The clarified lysate flowed through the column via a peristaltic pump. The column was then washed with EVNi1B Buffer (50 mM HEPES pH 7.5, 600 mM NaCl, 20 mM imidazole, 10% glycerol *w*/*v*). The His-tagged protein was slowly eluted over a linear gradient from EVNi2A (50 mM HEPES pH 7.5, 300 mM NaCl, 20 mM imidazole, 10% glycerol *w*/*v*) to EVNi2B (50 mM HEPES pH 7.5, 300 mM NaCl, 125 mM imidazole, 10% glycerol *w*/*v*) using an AKTA FPLC. The presence of EV68-3C protease in the elution peak was confirmed by SDS-PAGE. The His tag was then cleaved by addition of thrombin to the pooled fractions from the His-Trap crude FF purification, resulting in an authentic N-terminus. Cleavage proceeded overnight at 4 °C while dialyzing into SEC buffer (25 mM HEPES pH 7.5, 300 mM NaCl, 10% glycerol *w*/*v*). The cleaved, purified protein was collected by flowing over a 5 mL His-Trap crude FF column pre-equilibrated with EVNi1A buffer. The protein was concentrated to approximately 2 mL prior to purification via GFC on a Superdex 75 16/60 column. Fractions in the eluted peak were verified with SDS-PAGE, pooled, concentrated, and stored at –80 °C unless used immediately for crystallization.

### 2.2. Protein Crystallization

Purified EV68-3C protease, in the inactive form (C147A), and lyophilized substrate peptides purchased from GenScript (Piscataway, NJ, USA) for Biomedical Research were utilized in protein crystallization. Apo crystals of EV68-3C C147A and co-crystals of EV68-3C C147A with the 8-mer (TAKVQ|GPG) 3B3C substrate were produced according to conditions previously described by our group [42]. Crystals used for seeding were grown by thawing 10 mg/mL of protein on ice and diluting it to 5 mg/mL in SEC Buffer. Crystals were grown using 24-well, pre-greased, VDX hanging-drop trays (Hampton Research Corporation, Aliso Viejo, CA, USA) at various protein-to-precipitant ratios (1 μL:2 μL, 2 μL:2 μL, and 3 μL:2 μL) with 10–25% (*w*/*v*) PEG 3350, 0.10–0.20 M potassium thiocyanate, and 0.1 M Bis-Tris-Propane pH 6.5. The crystals grew over one week and due to their small size were used for seeding. Larger crystals were grown with seeding and by thawing 10 mg/mL of protein on ice, then diluting it to 7 mg/mL. These larger crystals were grown using 24-well, pre-greased, VDX hanging-drop trays (Hampton Research Corporation) at a protein-to-precipitant ratio (2 μL:2 μL with seeding, and 2 μL:2 μL without seeding) with 12–30% (*w*/*v*) PEG 3350, 0.20 M potassium thiocyanate, and 0.1 M Bis-Tris-Propane pH 6.5. Crystal growth took place at room temperature and required one week to obtain diffraction quality crystals for apo structure and several months for co-crystals that contained peptide. Prior to complex formation, the protein was centrifuged at 13,000× *g* for two minutes at 4 °C to remove the insoluble particulates that may promote aggregation and hinder crystal growth. All substrate complexes were formed by incubating EV68-3C with 10-fold molar excess of the 3B3C substrate peptide on ice for two hours. To limit vibration, crystallization trays were placed on foam padding.

### 2.3. Data Collection and Structure Determination

X-ray diffraction data was collected at 100 K. Co-crystals were soaked in cryogenic solutions made by supplementing the exact precipitant solutions with 15% ethylene glycol. Crystallographic data was collected locally at the University of Massachusetts Chan Medical School Crystallography and Structure-Based Drug Design Core facility and at the Brookhaven National Laboratory NSLS-II Beamline 17-ID-2 (FMX). In-house data collection was performed with a Rigaku MicroMax-007HF X-ray generator with a HyPix-6000HE detector. Diffraction data was indexed, integrated, and scaled using CrysAlisproPX (Rigaku Corporation, Tokyo, Japan). NSLS-II-collected diffraction intensities were automatically indexed, integrated, and scaled using XDS [50]. All structures were determined using molecular replacement with PHASER [51]. Model building and refinement were performed using Coot [52,53] and Phenix [54]. The reference model used to solve the apo EV68-3C protease by molecular replacement was PDB 7L8H [42]. The reference model used to solve the 3B3C co-crystal structure was the apo EV68-3C protease C147A mutant structure (PDB 8FL5). Prior to molecular replacement, the model was modified by removing all water, buffer, and cryogenic molecules as well as any small molecule inhibitors in the active site. To minimize reference model bias, 5% of the data was reserved to calculate R_free_ [55]. X-ray data collection parameters and refinement statistics are presented in Table 1.

### 2.4. Molecular Modeling

The substrates were modeled into the EV68-3C protease active site through alignment with the substrate-bound crystal structures of the 3C-like domain of SARS-CoV-2 main protease in PyMOL (version 2.07). Initially, EV68-3C protease bound to SG-85 (PDB ID: 3ZVF) was aligned to EV68-3C protease bound to AG7088 (PDB: 7L8H). From here, SARS-CoV-2 main protease bound to AG7088 (PDB: 7LHI) was aligned onto the EV68-3C protease bound to AG7088. With these three structures aligned, one final structure, SARS-CoV-2 main protease bound to nsp9-nsp10 (PDB: 7TA4), was then aligned to the AG7088 inhibitor bound to the SARS-CoV-2 main protease structure to inform peptide placement within EV68-3C protease. Using this information, the conserved P1 glutamine of the nsp9-nsp10 peptide was aligned to the gamma-lactam ring of SG-85 such that the P5 to P1 backbone could then be modeled against the backbone of the peptidomimetic inhibitor. The resulting peptide was mutated to the appropriate sequence of the EV68-3C 3B3C natural substrate (TAKVQGPGF) within PyMOL (version 2.07) and optimized as the starting model. All substrate peptides were modeled with the conserved glutamine at the S1 subsite. The initial models for the remaining peptides were generated by mutating the optimized 3B3C natural substrate to the corresponding sequences (Figure 1C).

### 2.5. Molecular Dynamics Simulations

A high-resolution crystal structure of EV68-3C protease (PDB 3ZVF) with the modeled peptide was prepared via the Protein Preparation Wizard within Schrödinger Suite [56] software Maestro (version 12.2.012) as previously described [45,57]. Missing side chains were added through Prime [58], hydrogen atoms were added to the structure, protonation states were determined via PROpKA [59], and the hydrogen bonding network was then optimized. Finally, a restrained minimization was performed using the OPLS3 force field [60] within an RMSD of 0.3 Å to minimize the structure prior to simulation. The catalytic histidine, H40, was deprotonated for all system preparations as the predicted pKa (6.49) was below 7.0. The prepared systems were placed in a cubic TIP3P explicit water box measuring 25 Å on each side. MD simulations were carried out as previously described [57] using Desmond within the Schrödinger Suite [56]. To neutralize the system, 0.15 M salt was added using sodium cations and chloride anions to the system. The OPLS3 force field was used to parametrize the substrate and protein. All crystallographic waters were retained during structure minimization and the following MD simulation. Prior to starting the 150 ns MD simulations, the solvated system was minimized using a gradual, 21 stepwise procedure, similar to a prior 14 stepwise procedure described previously [57], but with additional steps with smaller incrementation. Triplicates of 150 ns simulations for EV68-3C protease with each modeled substrate and randomized velocity were started using a protocol previously developed [45,57]. The root-mean-square deviation (RMSD) and root-mean-square fluctuations (RMSFs) of alpha carbon atoms were calculated using tools within the Schrödinger Suite as well as through previously described custom python scripts on github [61].

### 2.6. Structural Analysis: Hydrogen Bonds

The co-crystal structures contained two EV68-3C protease monomers and the 3B3C peptide within the active site in the asymmetric unit. For these complexes with two monomers in the asymmetric unit, the protease chain with clear electron density around the active site and substrate (chain C) was chosen for analysis. For the modeled structures, chain B was chosen for the 2A2B, 2B2C, 2C3A, 3A3B, 3B3C, 3C3D, VP0VP3, and VP3VP1 substrates.

For all structures, intermolecular interactions were analyzed using custom python scripts as previously described [61]. Hydrogen bonds were displayed using the show contacts PyMOL Plugin with default parameters where the bond angle is between 63° and 180° and the distance is less than 4.0 Å for any and 3.6 Å for an ideal hydrogen bond between the proton and heavy atom. Further analysis was conducted on simulation trajectories to determine the high-frequency hydrogen bonds between the substrate and protease. Van der Waals interaction energies between the inhibitor and protease were estimated using a simplified Lennard–Jones potential, as previously described [62].

### 2.7. Substrate Envelope

To define the substrate envelope [43], the modeled structures were superimposed using the carbon alpha atoms of active site residues and highly stable residues as determined by distance difference calculations (residues 40, 126, 142, 144, 146, 147, 152, 157, 158, 161, 162). After superimposition, a Gaussian object map was generated for each substrate where the van der Waals volume was mapped onto a grid with a spacing of 0.5 Å. In addition, the MD simulation trajectories were used to calculate the dynamic substrate envelope. Utilizing all converged frames from the 150 ns MD simulations, the dynamic intersecting volumes for all eight substrates were calculated. Summation of these maps generated the consensus volume occupied by all substrates, which was used to construct the substrate envelope. The figures were generated using PyMOL and Maestro (Schrödinger) to visualize the substrate envelopes [56].

## 3. Results

### 3.1. Binding of Substrates to EV68-3C Protease: Modeling and Validation

To determine the substrate envelope of EV68-3C protease, peptides with amino acid sequences corresponding to the eight natural cleavage sites in the viral polyprotein were modeled within the protease active site. A high-resolution co-crystal structure of the protease with a peptidomimetic inhibitor (PDB ID: 3ZVF; Figure 1B) was chosen as the starting structure. This inhibitor, SG-85, guided the modeling of the peptide, especially the backbone, within the active site from the P4 to P1′ positions [17]. The prime side positions beyond P1′ were modeled based on our experimental crystal structure of SARS-CoV-2 3C-like main protease bound to the most evolutionarily conserved substrate sequence, nsp9-nsp10 (PDB ID: 7TA4) [23]. We found that peptides containing eight residues spanning P5 to P3′ were optimal, as the more distal P4′ and P5′ positions did not have considerable interactions with the protease. Thus, leveraging the available structural information from these co-crystal structures, we generated eight models of substrate complexes of EV68-3C protease.

Molecular dynamics (MD) simulations were performed on all eight protease–peptide complexes (see Methods) in triplicate for 150 ns to both validate the generated models and compare the binding of substrates. These were stable simulations as judged by the RMSF values of both the protein and the bound substrate peptide (Figure S1). The lowest fluctuations were centered around the P1 glutamine, with an average of 0.3 Å, and were below 1 Å deviation from P4 to P2′. Greater fluctuations were observed at the termini, P5 and P3′, as expected. With low fluctuations and conserved binding modes throughout the simulations, these simulations provided additional support that the modeled peptides were stable within the active site.

### 3.2. Conserved Hydrogen Bonding between the Substrates and Protease

**Figure 2:**
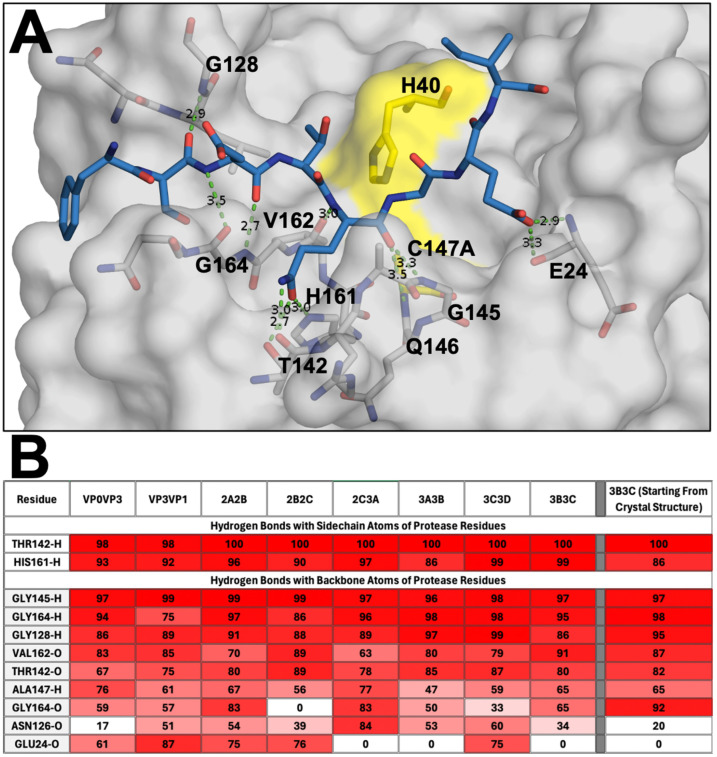
Hydrogen bond network between the substrate peptides and the active site of EV68-3C protease. (**A**) The EV68-3C protease in grey surface representation, focusing on the active site pocket with the catalytic dyad highlighted in yellow, and the end point modeled 3C3D peptide (8 mer) post MD simulation shown in blue sticks. Hydrogen bonds between the protein and peptide heavy atoms are depicted with green dashed lines. (**B**) Percent frequency of the hydrogen bonds between EV68-3C protease and modeled substrate peptides, as well as the 3B3C peptide co-crystal structure (PDB ID: 9AX9), during MD simulation trajectories. The bonds present during the simulation with higher frequency interactions are denoted in darker red and lower frequency interactions are in white.

The hydrogen bonds between the modeled peptides and EV68-3C protease active site residues throughout the MD simulation trajectories were analyzed to evaluate the intermolecular interactions. Despite diversity in sequence among the substrate peptides, nine hydrogen bonds involving the backbone atoms of the peptide as well as two specific sidechains were conserved for all complexes (Figure 2). Overall, these hydrogen bonds were analogous to those observed in the co-crystal structures of substrate peptides with SARS-CoV-2 main protease [23]. Most, if not all, of these hydrogen bonds (Figure 2B) were highly stable and conserved during the simulations, supporting the validity of our molecular modeling.

Within this network of hydrogen bonds, we found that residues Thr142 and His161 of EV68 stabilized the P1 glutamine across all modeled peptides within the S1 pocket. Specifically, these conserved interactions formed between the following: Thr142 sidechain hydroxyl hydrogen with the P1 glutamine sidechain carbonyl (99.5%), Thr142 backbone carbonyl with the P1 glutamine sidechain amine (80.1%), and His161 ring’s tau-nitrogen with the P1 glutamine sidechain carbonyl (94.0%). Near the catalytic residues, the Gly145 backbone nitrogen interacted with the P1 backbone carbonyl, forming an extremely stable hydrogen bond, present in over 96% of the simulation trajectory for all peptides. This same P1 backbone carbonyl can also hydrogen bond with the backbone nitrogen of residue 147, the catalytic cysteine (63.5%). The P1 glutamine was further stabilized by a hydrogen bond between the backbone carbonyl of Val162 and the backbone nitrogen of the P1 glutamine (80.0%). These conserved interactions with the protein further contribute to stabilize the glutamine in the S1 pocket of the active site.

Distal to the P1 glutamine, other interactions were highly conserved. Gly164 formed extensive hydrogen bonds with the P3 residues to stabilize the peptide. At this position, the Gly164 hydrogen bonds through its backbone nitrogen to the backbone carbonyl of the P3 residue (93.4%). While less frequent, the backbone carbonyl of Gly164 also had a conserved hydrogen bond with the backbone nitrogen of the P3 residue (53.8%). Within the slightly flexible loop 125-129, the backbone nitrogen of Gly128 formed a hydrogen bond with the P4 backbone carbonyl group (90.6%). This interaction with the P4 backbone of the peptide improved the stability of the distal P side residues. On the P′ side, the P2′ sidechain can often form a hydrogen bond with either the backbone carbonyl or backbone nitrogen of Glu24.

### 3.3. Substrate Envelope of EV68-3C Protease

**Figure 3:**
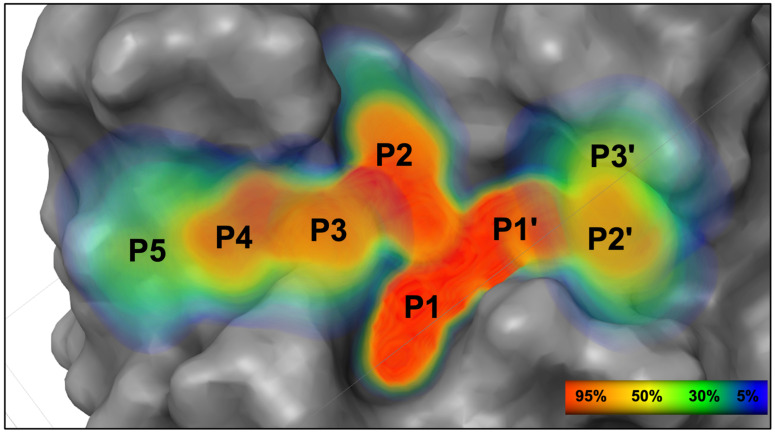
The substrate envelope of EV68-3C protease. A heatmap representation of the calculated dynamic substrate envelope for EV68-3C protease shows the percent occupancy of viral substrates within this three-dimensional volume. The peptide backbone is most well conserved between the P4 and P2′ positions and has more variability at P5 and at P3′. The percent occupancy ranges from lowest (dark blue) to highest (red) of finding atoms at that position within a given viral peptide.

The conserved volume occupied by the diverse substrates when bound at the protease active site was determined, defining the EV68-3C protease substrate envelope. For each substrate, the MD simulations were used to calculate the volume occupied throughout the replicate trajectories. The structures from the simulations were superimposed using invariable alpha carbon atoms (see Methods). After superposition, the van der Waals volume of the substrate was mapped onto a grid to calculate a Gaussian object map for the occupancies of grid cells throughout the simulations, as we have conducted previously [48,49]. Summation of these maps for all eight substrates defined the conserved volume occupied, thus the substrate envelope (Figure 3). The position of the peptide backbones of all substrates were conserved with a greater than 95% occupancy (shown in red) from P4 to P2′ as were the sidechains from P2 to P1′. Despite the high diversity among amino acid sequences, this highly conserved space from P4 to P2′ suggests an evolutionarily constrained portion of the EV68-3C protease active site. Overall, the stability of the modeled peptides and the resulting substrate envelope suggested an evolutionarily conserved three-dimensional shape underlying substrate recognition by EV68-3C protease.

### 3.4. Crystal Structure of EV68-3C Protease with a Bound Peptide Validates the Substrate Envelope

**Figure 4:**
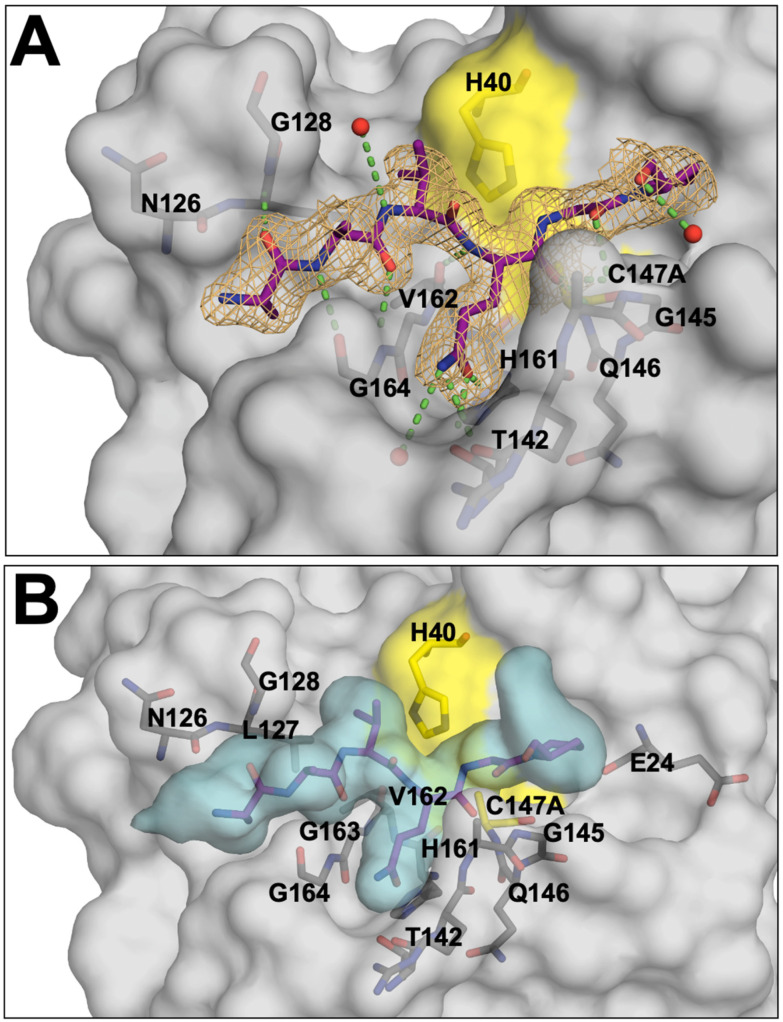
The experimental crystal structure of EV68-3C protease with a substrate peptide bound at the active site. (**A**) The EV68-3C protease (grey surface) co-crystal structure with the 3B3C peptide (purple sticks) in the electron density, with nearby crystallographic waters (red spheres) and hydrogen bond interactions (green dashed lines) with the surrounding protease residues (dark grey sticks). The mesh depicts the electron density (2F0-FC map) for the 3B3C peptide at the active site. (**B**) The EV68-3C protease (grey surface) and 3B3C peptide (purple sticks) co-crystal structure shown with the superimposed substrate envelope denoted by the cyan volume. The peptide, derived from an experimental protein crystal structure, fits completely within the calculated envelope from molecular modeling and MD simulations.

While the co-crystal structures of EV68-3C protease with viral substrates had remained elusive, we have solved and refined the first such co-crystal structure. The protease was inactivated by mutating the catalytic cysteine into an alanine, C147A, to prevent substrate cleavage while crystallizing with an 8-mer peptide corresponding to the 3B3C cleavage site. This structure was determined to 2.0 Å resolution with an R_factor_ of 18.08% and R_free_ of 22.82% (PDB ID: 9AX9), as shown in Table 1. This structure resolved the bound peptide from the P4 to P2′ positions (Figure 4A) in the electron density. We also solved and refined the crystal structure of the apo inactive protease to 1.8 Å resolution with an R_factor_ of 17.44.% and R_free_ 24.54% (PDB: 8FL5), as shown in Table 1. These experimental crystal structures provided us with important information to validate the molecular models and simulations. The experimental co-crystal structure with the 3B3C peptide was analyzed and compared to our molecular model. Superposition of these two structures, experimental and computational, revealed a very good agreement with the converged modeled substrate peptide position (Figure S2), and the peptide from the experimental crystal structure fits well within the substrate envelope (Figure 4B). The crystal structure of the protease with the 3B3C peptide was subjected to the same MD simulation protocol to further validate the model. From these MD simulation trajectories, we analyzed the hydrogen bonding network. The hydrogen bonds observed in our molecular model simulations were maintained during the MD simulation of the 3B3C co-crystal structure, with nearly identical frequencies (Figure 2B). To further quantify the agreement of the experimental structure with the model, the Pearson correlation for the hydrogen bond frequencies at the conserved residues was calculated as 0.95 with an R-squared value of 0.89. These results further supported our molecular modeling approach, validating peptide positioning within the active site and the conformations used to determine the substrate envelope.

### 3.5. Substrate-Induced Conformational Changes of the Protease

**Figure 5:**
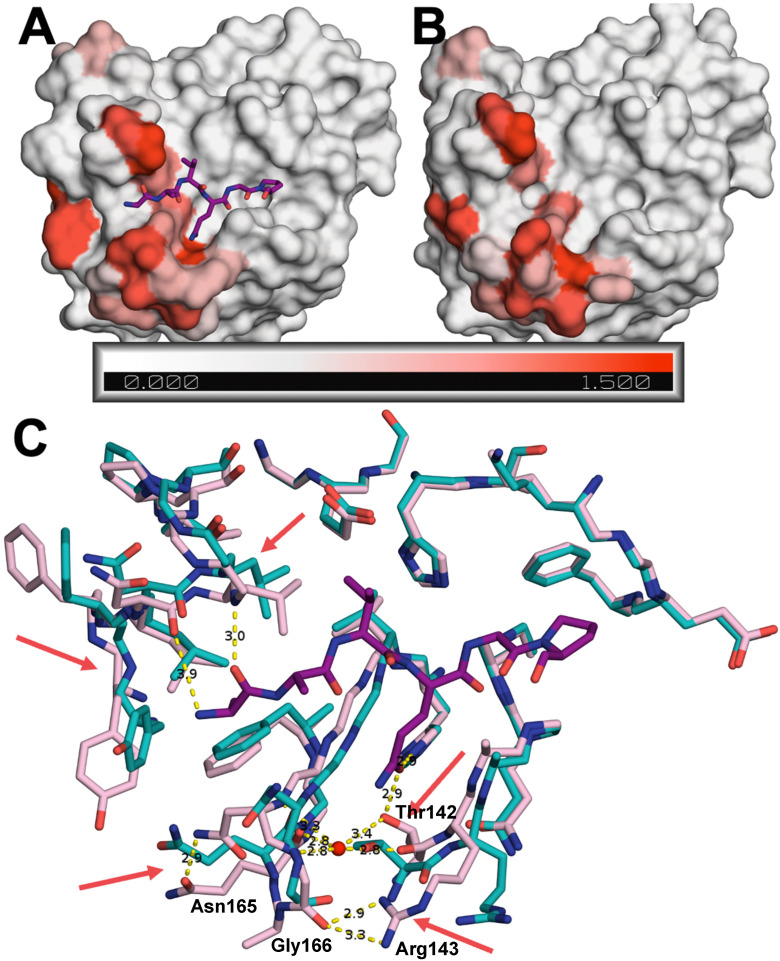
Comparison of peptide-bound and apo EV68-3V protease crystal structures. Surface representation of (**A**) 3B3C-peptide-bound (PDB ID: 9AX9) and (**B**) apo (PDB ID: 8FL5) protease colored according to variation of backbone, assessed by the average difference in distance between the C-alpha atoms of residues (in Å) between the two structures. (**C**) Superposition of the two structures in stick representation with red arrows pointing to the largest changes in key residues between the apo (cyan) and the substrate-bound (pink) co-crystal structures. The 3B3C peptide is depicted as purple sticks.

The crystal structures were determined for the EV68-3C protease with (PDB ID: 9AX9) and without a substrate bound at the active site (PDB ID: 8FL5). These two structures were determined in the same space group, with similar cell dimensions and resolution. This allowed us to directly compare the two structures and identify any conformational changes induced upon substrate binding (Figure 5). The C-alpha distances between the two structures were calculated and the average for each residue mapped onto the two structures (ranging from 0 Å to 1.5 Å). The largest variation was observed around the active site, at the P1 binding pocket. In the apo structure, the areas surrounding the P1 glutamine binding pocket form a narrow and more open channel (Figure 5B). However, once the peptide binds, the pocket becomes capped off, enclosing the P1 glutamine peptide in a U-shaped pocket (Figure 5A). Specifically, the Thr142 sidechain is shifted 3.6 Å deeper into the S1 pocket, opening that area for a water molecule to coordinate with the backbones of Gln168, Gly166, and Asn165 (Figure 5C). At the same time, Thr142 is positioned closer to the P1 glutamine, enabling the Thr142 hydroxyl to form a hydrogen bond with the incoming substrate peptide. Proximal to the structural water, we observed a 4.3 Å conformational change of Arg143 to form a hydrogen bond with Gly166 that is unique to the substrate-bound structure forming the S1 pocket.

Compared to the apo protease, loop 125–129 retained its overall shape but was closer to the P3 and P4 region of the substrate in the bound structure. For the substrate-bound protease, Asn126 in this loop was 0.7 Å closer to the peptide, enabling a hydrogen bond. Similarly, Gly128 was 1.3 Å closer to the P4 backbone nitrogen in the substrate-bound structure, facilitating another hydrogen bond interaction. Thus, rearrangements altered the overall shape of the active site to enable hydrogen bonding interactions with the substrate peptide.

### 3.6. Potential Sites for Inhibitor-Induced Resistance Mutations of the EV68-3C Protease

**Figure 6:**
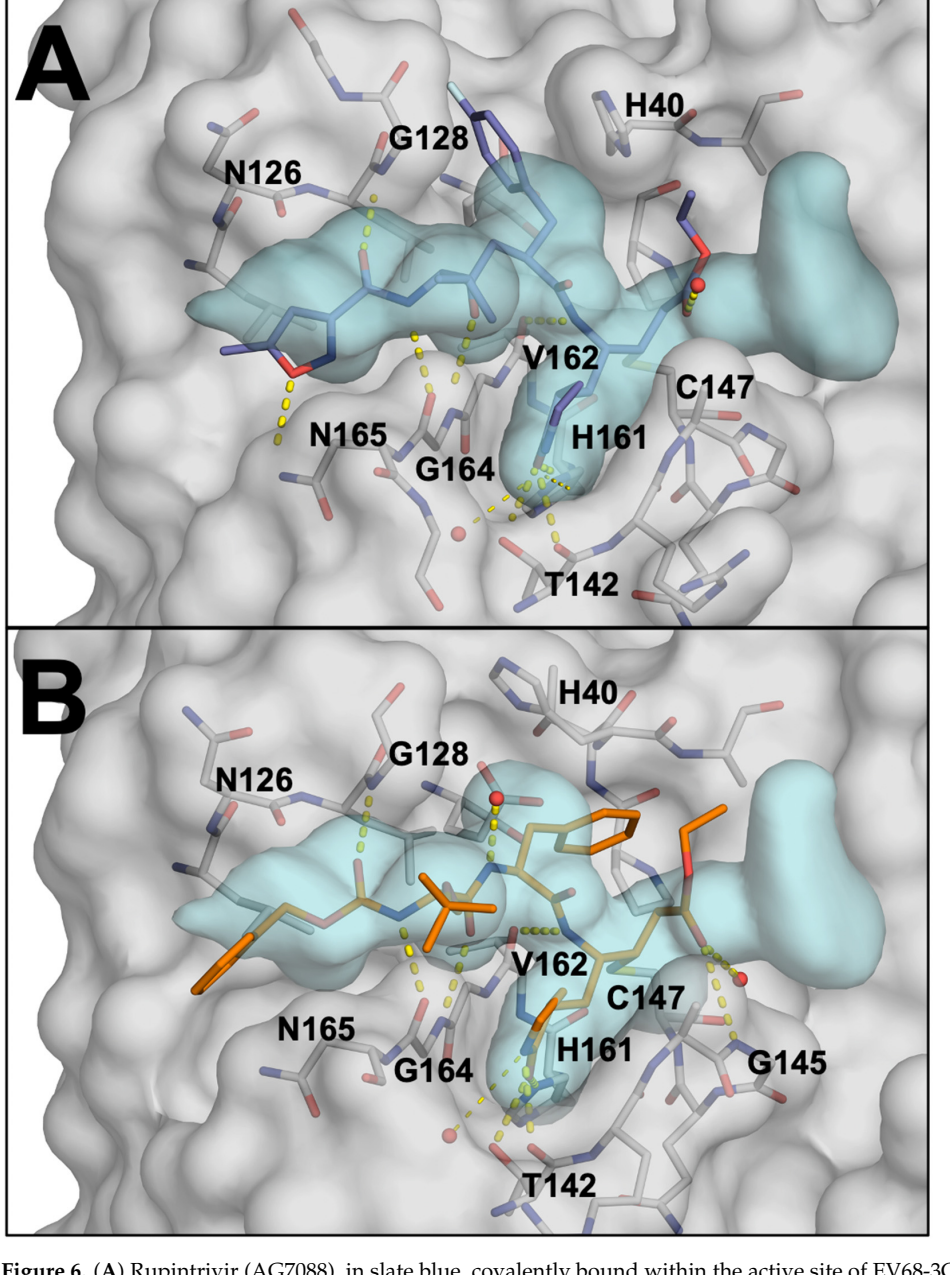
(**A**) Rupintrivir (AG7088), in slate blue, covalently bound within the active site of EV68-3C protease in grey (PDB: 7L8H). Hydrogen bonds between rupintrivir and the protein are highlighted as yellow dashed lines. The substrate envelope is shown in light blue (**B**) SG-85, in orange, covalently bound within the active site of EV68-3C protease (PDB: 3ZVF).

Determining the substrate envelope of EV68-3C protease enabled us to identify where inhibitors protrude beyond the envelope and thereby evaluate their susceptibility to resistance. Inhibitors of EV68-3C protease include irreversible covalent inhibitors. These inhibitors, such as rupintrivir (AG7088) and a suite of peptidomimetics, have been previously co-crystallized with EV68-3C protease [17,42]. To evaluate potential sites of resistance to rupintrivir (PDB ID: 7L8H) (Figure 6A) and SG-85 (PDB ID: 3ZVF) (Figure 6B), inhibitor fit was analyzed within our EV68-3C substrate envelope. Both inhibitors protrude out of the substrate envelope at the S1′, S1, S2, and S5 positions to varying degrees. Resistance mutations are less likely at the S1′ pocket, due to packing of the inhibitor on the active site residues His40 and Cys147 which cannot mutate without obliterating activity. However, mutations at Ala144 or Gly166 could potentially introduce steric bulk to block the protruding P1 moieties. Similarly, larger residues introduced at Gly128 could interfere with the binding of P2 rings to confer resistance. At the S5 pocket, mutation of Asn165 to a larger residue could preclude binding of the inhibitors. Thus, for both inhibitors, by evaluating the substrate envelope, we have identified potential sites for resistance mutations to occur.

## 4. Discussion

With the continued emergence of new EV68 variants in the United States and long-term neurological sequalae associated with severe disease burden of infections, a better understanding and characterization of the virus and the conserved 3C protease is needed [11,12,13,14,15]. Targeting the conserved 3C proteases of various viral families is not a novel concept, and while 3C inhibitors have been developed previously [17,33,34,36,37,38,39,40,41], there is no FDA-approved treatment to address these enteroviral species. Furthermore, those inhibitors were not designed as robust compounds that account for possible resistance mutations, seeing up to a 15-fold decrease in potency when mutations arise [47]. To address this challenge, we successfully utilized molecular modeling, MD simulations, and protein crystallography to explore the EV68-3C protease active site, calculate the substrate envelope, uncover molecular interactions involved in substrate binding, as well as assess inhibitors for potential resistance mutations. With the apo and peptide-bound co-crystal structures with a viral cleavage site of the EV68-3C, we were also able to characterize the large conformational changes that occur upon substrate binding.

The substrate envelope applied to inhibitor-bound structures can provide insight into which residues of the protease could potentially mutate to confer resistance. When a portion of the inhibitor extends beyond the confines of the envelope, opportunity for resistance arises. When analyzing the potent 3C protease inhibitor, rupintrivir, there were potential sites for resistance mutations at the S1, S2, and S5 pockets at Ala144, Gly166, Gly128, and Asn165. In a previous study on HRV 3C protease, a single point mutation at the analogous position of Asn165 was reported to confer a five-fold decrease in inhibitor potency, with additional mutations (A103V, E3G) decreasing potency up to 15-fold [47]. As the HRV and EV68-3C proteases are well conserved, it is likely that a similar resistance profile could occur for a single Asn165 point mutation or a triple mutant (N165T, A103V, E3G) in EV68-3C protease. Assessing the impact of these mutations, before resistance arises, is key to the development of long-lasting and robust EV68-3C inhibitors.

EV68 continues to present as a global health concern with severe cases resulting in long-lasting neurological deficits and death as there are currently no FDA-approved antiviral drugs for treatment. Through this work, we have characterized an evolutionarily conserved viral protein, 3C protease, also found in several related viruses. Not only does this work provide the foundation for designing improved therapeutics against EV68, but this strategy can also apply to other enteroviruses, such as EV71 or EV93, human rhinovirus, coxsackies virus, and others. As these viruses mutate, generating new strains, the potential for more dangerous strains and formation of resistance remains a threat to health. Beyond natural evolution, inhibitors also apply selective pressure, further accelerating the emergence of resistance mutations. We have previously shown that emergence of resistance can be minimized by designing inhibitors that leverage the evolutionary constraint of substrate specificity. Here, we characterized that substrate specificity, which will enable the rational design of novel therapeutics, resilient to resistance mutations of EV68-3C protease. These same techniques and methodology can be extended to other viruses to characterize 3C proteases and evaluate inhibitors with the goal of developing robust therapeutics.

## Figures and Tables

**Table 1:** Crystallographic data collection and refinement statistics for EV68-3C protease apo and 3B3C substrate peptide-bound structures.

	EV68-3C apo	EV68-3C–3B3C
PDB ID	8FL5	9AX9
**DATA COLLECTION**		
Location	Home Source	NLS-II, Synchrotron
Resolution Range (Å)	23.74–1.8 (1.864–1.8)	30.19–2.00 (2.07–2.00)
Space Group	P 1 21 1	P 1 21 1
a,b,c, (Å)	39.1656, 102.109, 41.9459	39.784, 103.343, 43.213
alpha, beta, gamma (°)	90, 109.88, 90	90, 110.975, 90
Total Reflections	116,426 (8510)	42,866 (4233)
Unique Reflections	28,677 (2867)	21,924 (2200)
Multiplicity	4.1 (3.0)	2.0 (1.9)
Completeness (%)	99.9 (99.7)	99.5 (100.0)
(Average I)/sigma	14.3 (1.6)	15.0 (4.3)
Wilson B-Factor	25.33	32.08
R_merge_	0.0718 (0.577)	0.029 (0.163)
CC1/2	0.997 (0.646)	0.998 (0.942)
**REFINEMENT**		
R_factor_	0.1744 (0.2608)	0.1808 (0.2233)
R_free_	0.2454 (0.3337)	0.2282 (0.3062)
**RMSD in:**		
Bond Lengths (Å)	0.011	0.02
Bond Angles (°)	1.2	0.48
**Ramachandran:**		
Favored (%)	95.57	95.58
Allowed (%)	4.43	4.14
Outliers (%)	0	0.28
Rotamer outliers (%)	0.35	1.09
**B-Factors:**		
Average	30.61	39.65
Macromolecules	29.87	39.03
Solvent	37.05	47.18
Peptide	NA	53.44

R_factor_ = Σ || F_o_| − |F_c_||/Σ|F_o_| and R_free_ values were calculated from 5% of reflections, chosen randomly, which were omitted from the refinement process. Statistics for the highest resolution shell are shown in parentheses.

## Data Availability

The data that support this study are available from the corresponding authors upon reasonable request. The crystal structures determined in the current study are available in the Protein Data Bank (https://www.rcsb.org) with accession codes 8FL5 and 9AX9.

## Supplementary Materials

The following supporting information can be downloaded at: https://www.mdpi.com/article/10.3390/v16091419/s1, Figure S1: Peptide backbone fluctuations (RMSF) during simulations of molecular models; Figure S2: Superposition of model and crystal structure of 3B3C peptide bound to EV68-3C protease.
