# Supplementary material for: Elucidating the Substrate Envelope of Enterovirus 68-3C Protease: Structural Basis of Specificity and Potential Resistance
Source: Viruses. 2024 Sep 5;16(9):1419. doi: 10.3390/v16091419 (PMC11437433; doi:10.3390/v16091419)
Supplement: Supplementary file 1 [file viruses-16-01419-s001.zip › viruses-3186739-supplementary.pdf]

Supplementary Materials

Elucidating the Substrate Envelope of Enterovirus 68-3C Protease: Structural Basis of Specificity and Potential Resistance

Vincent N. Azzolino, Ala M. Shaqra, Akbar Ali, Nese Kurt Yilmaz and Celia A. Schiffer *

Department of Biochemistry and Molecular Biotechnology, University of Massachusetts Chan Medical School, Worcester, MA 01605, USA; vincent.azzolino@umassmed.edu (V.N.A.); ala.shaqra@umassmed.edu (A.M.S.); akbar.ali@umassmed.edu (A.A.); nese.kurtyilmaz@umassmed.edu (N.K.Y.)

* Correspondence: celia.schiffer@umassmed.edu

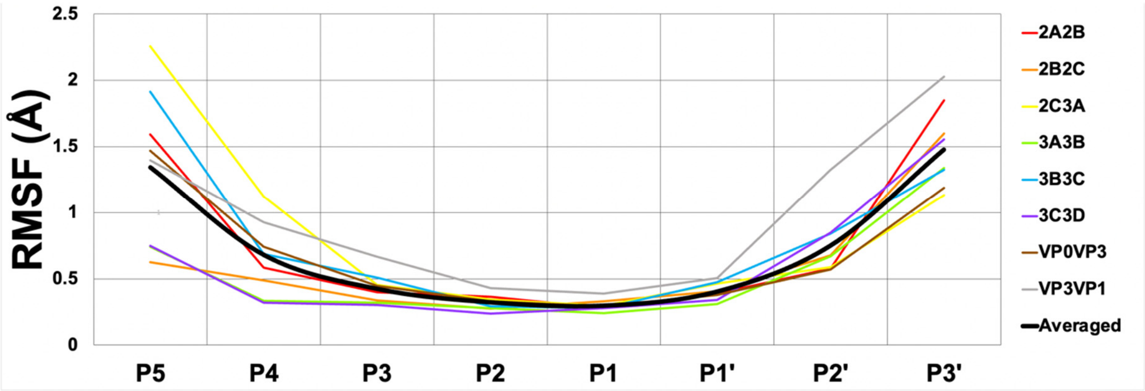

Figure S1. Substrate peptide fluctuations during 150 ns molecular dynamics (MD) simulations of molecular models bound to EV68-3C protease. The root-mean-squared fluctuations (RMSF) of viral substrate backbone were calculated (colored lines), and then averaged at each position (bolded black line).

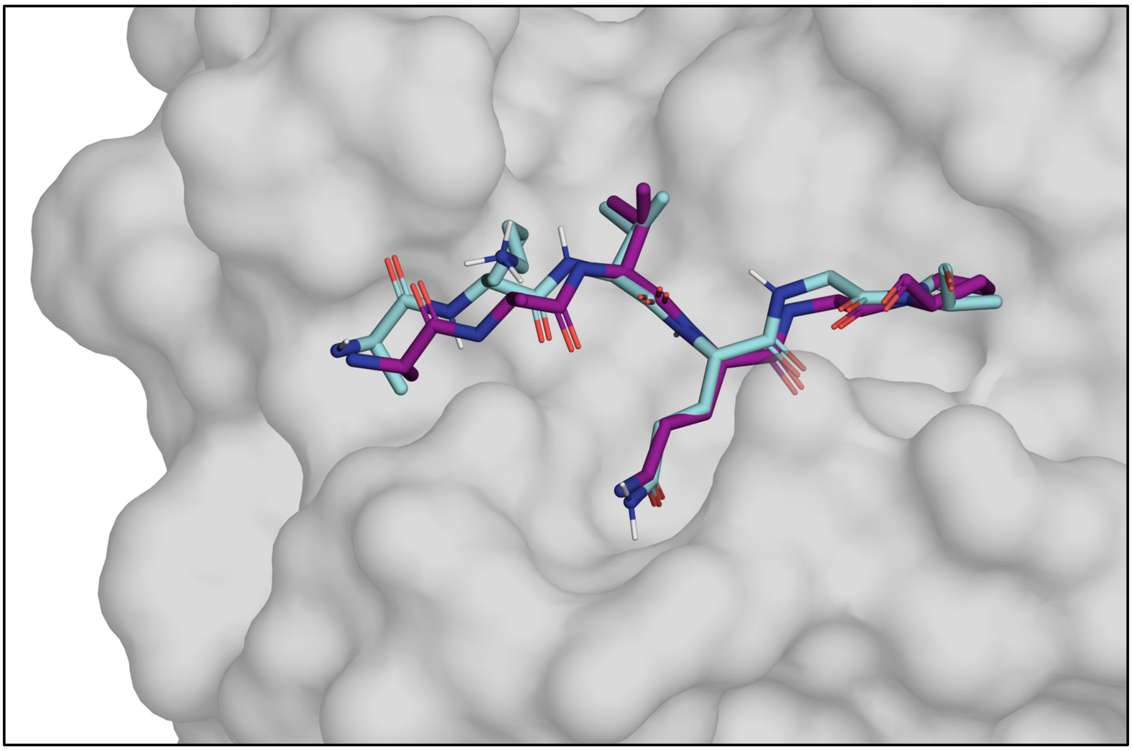

Figure S2. EV68-3C protease bound to the 3B3C peptide from the molecular model (cyan) compared with the experimental co-crystal structure (purple). The agreement between the two structures further supports and validates the molecular modeling approach. The protease is shown in gray surface representation.
